# Effect of Normothermic Machine Perfusion on Glycocalyx Shedding During Liver Transplantation – A Prospective Pilot Study

**DOI:** 10.3389/ti.2026.15502

**Published:** 2026-03-02

**Authors:** Simon Mathis, Gabriel Putzer, Lukas Gasteiger, Nikolai Staier, Lisa Schlosser, Pia Tscholl, Robert Breitkopf, Benno Cardini, Alexander Kofler, Rupert Oberhuber, Thomas Resch, Stefan Schneeberger, Judith Martini

**Affiliations:** 1 Department of Anaesthesiology and Critical Care Medicine, Medical University of Innsbruck, Innsbruck, Austria; 2 Department of Visceral, Transplant and Thoracic Surgery, Medical University of Innsbruck, Innsbruck, Austria; 3 Data Lab Hell GmbH, Zirl, Austria

**Keywords:** glycocalyx, heparan sulfate, liver transplantation, normothermic machine perfusion, syndecan-1

## Abstract

Ischemia–reperfusion injury (IRI) plays a pivotal role in liver transplantation by inducing oxidative stress and inflammation, thereby contributing to impaired graft function and postoperative complications. A key element of IRI is degradation of the endothelial glycocalyx, resulting in microcirculatory dysfunction. This study investigated the impact of normothermic machine perfusion (NMP) on glycocalyx integrity and its association with early postoperative outcomes. Thirty grafts undergoing NMP prior to transplantation were analyzed. Syndecan-1 and heparan sulfate were quantified in perfusate and recipient serum. Donor-related factors influencing glycocalyx injury during NMP were assessed, and correlations with outcomes established. Syndecan-1 levels increased during NMP and remained significantly elevated in grafts from circulatory-death (DCD) donors compared with brain-death (DBD) donors. Receiver operating characteristics revealed predictive potential for early allograft dysfunction (EAD) with a syndecan-1 cut-off of 4,796.13 ng/mL after 6 h of NMP. In contrast, heparan sulfate concentrations showed no relevant changes. Postoperatively, syndecan-1 levels in recipient serum were elevated immediately after transplantation but declined over subsequent days, while heparan sulfate remained stable. These findings indicate that glycocalyx injury develops during NMP, particularly in DCD livers, with elevated syndecan-1 reflecting endothelial vulnerability and a potentially modifiable aspect of graft physiology relevant to future protective strategies.

**Clinical Trial Registration:**

www.Clinicaltrials.gov, identifier NCT: 04764266.

## Graphical Abstract

**Figure:**
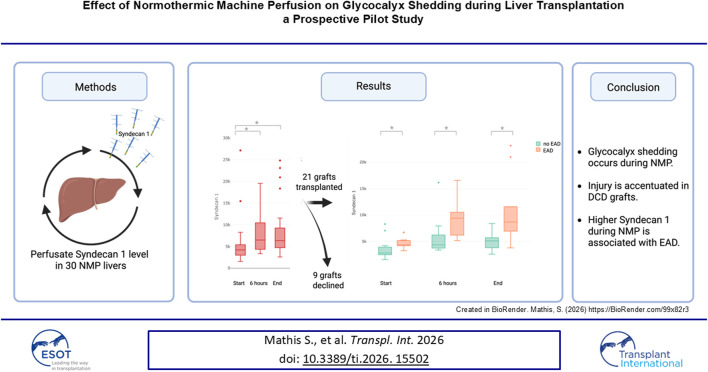

## Introduction

Ischemia-reperfusion injury (IRI) represents an unavoidable pathological process in liver transplantation, known to negatively affect graft function and patient outcome [1–3]. The abrupt restoration of blood flow after ischemic storage triggers a cascade of oxidative stress and inflammatory responses, characterized by the excessive production of reactive oxygen species (ROS) and the activation of immune pathways [4].

Probably one of the first structural damages in the onset of IRI, and certainly one of the key components of IRI is the disruption of the endothelial glycocalyx, a thin layer of proteoglycans, glycoproteins and glycosaminoglycans that lines the luminal surface of vascular endothelial cells [5–7]. During IRI, glycocalyx components, e.g., syndecan-1 and heparan sulfate are sheared from the endothelium and released into the blood. Glycocalyx shedding leads to microcirculatory disturbances, resulting in altered capillary perfusion and leukocyte-mediated tissue inflammation [8–10]. These microcirculatory disturbances have been shown to correlate with organ dysfunction after liver transplantation [11]. Microcirculatory dysfunction is aggravated by a reduction of shear stress mediated nitric oxide (NO) release, which further deteriorates capillary perfusion [12]. Although the exact pathomechanism is unclear, glycocalyx disruption also appears to be involved in the development of interstitial graft edema due to loss of barrier function and development of capillary leakage [13–15].

In recent years, normothermic machine perfusion (NMP) of the liver has emerged as a well-established procedure in many transplant centers. Beyond its recognized benefits for assessing organ function, NMP is gaining increasing attention for its potential role in mitigating IRI, as the process of organ reperfusion is shifted from the recipient to the perfusion device. This not only allows for better characterization of IRI but also has the potential to identify possible therapeutic targets. Studies showed that in contrast to static cold storage (SCS), NMP seems to mitigate IRI through multiple pathways [16–18]. Specifically, a shift in gene expression from a pro-inflammatory to a regenerative profile and a reduction in necrosis was observed in NMP liver grafts [18].

Although glycocalyx damage after SCS has been the focus of numerous studies, the extend of glycocalyx damage during NMP remains to be elucidated. To investigate this, we analyzed glycocalyx alterations during NMP, with a specific focus on identifying donor characteristics that may contribute to glycocalyx degradation [19, 20]. Additionally, we aimed to evaluate whether changes in glycocalyx integrity influence postoperative outcomes in liver transplant recipients.

## Materials and Methods

In this study, 30 livers from deceased donors subjected to NMP were included after the allocated recipients of the organs provided written informed consent to participate in the study. The study was approved by the local ethics committee (approval number 1382/2020) and registered with ClinicalTrials.gov (www.Clinicaltrials.gov; NCT: 04764266, Simon Mathis MD; February 17th, 2021). It was conducted in accordance with the Declarations of Helsinki and Istanbul. Grafts allocated to patients younger than 18 years of age and organs allocated to patients undergoing re-transplantation were excluded.

The primary endpoint of the study was the temporal profile of the glycocalyx components syndecan-1 and heparan sulfate in the perfusate during normothermic machine perfusion. Secondary endpoints included donor factors influencing the extent of glycocalyx injury during NMP; in transplanted grafts, the temporal profile of glycocalyx components in the recipient and associations between the extent of glycocalyx injury and recipient outcome were analyzed as secondary endpoints.

### NMP Data

NMP for the livers was initiated using the OrganOx metra® liver perfusion device (OrganOx, Oxford, UK) in a back-to-base approach as previously described [21–23]. The perfusion fluid consisted of 840 mL packed red cells and 500 mL gelatin solution (Gelofusin®, B. Braun, Melsungen, Germany). In addition, 10,000 IU heparin, calcium gluconate and sodium bicarbonate were added to the priming. Measurements of the glycocalyx shedding parameters syndecan-1 and heparan sulfate were performed at three time points during NMP: Immediately after initiation of NMP (NMP 1), after 6 h of NMP (NMP 2) and right before the end of NMP (NMP 3). For each time point, 4.5 mL of perfusate was collected, centrifuged and stored at −80 °C until analysis. Perfusate electrolyte and metabolic composition were assessed at the same time points. The decision to accept or reject a graft for transplantation during NMP was at the discretion of the transplant surgeon on duty based on local SOP [24]. Even if the graft was declined for transplantation, the data collected during NMP were included in the analysis.

### Donor and Recipient Data

Donor characteristics (age, sex, weight, donor type), graft characteristics (SCS-time, NMP-time) and recipient demographics (age, sex, weight, height, Meld score, etiology of liver disease) were collected.

Liver transplantation was performed according to local standard operating procedure using the total cava replacement technique or the piggyback technique without the use of a cavo-caval shunt.

Postoperative data were collected daily during the first seven postoperative days and included bilirubin, aspartate aminotransferase (AST), alanine aminotransferase (ALT), glutamyl transferase (GGT), alkaline phosphatase, acetyl cholinesterase, c-reactive protein (CRP), procalcitonin and coagulation parameters (PT, aPTT, fibrinogen). In addition, the need for renal replacement therapy, occurrence of early allograft dysfunction (EAD), length of ICU stay, 30-day and 90-day mortality were recorded [25].

To assess glycocalyx degradation in the recipient, 4.5 mL of EDTA blood was collected from the recipient before induction of anesthesia [Baseline (BL)], on admission to the intensive care unit (ICU) and on postoperative days 1 (D1), 2 (D2) and 3 (D3). All samples were centrifuged and frozen at −80 °C until subsequent analysis. Syndecan-1 concentrations were measured in perfusate and patient plasma by Human CD138 ELISA KIT, Diaclone SAS, Besancon Cedex, France; heparan sulfate levels were determined using Human Heparan Sulfate Proteoglycan (HSPG) ELISA KIT, Reddot Biotech inc., Kelowna, Canada.

### Statistics

Sample size estimation for assessing glycocalyx changes during NMP was based on data from two previously published studies [19, 22]. Assumptions were made regarding a statistical power of 80% and a significance level of 5%.

Statistical analysis was performed using “R” (version 4.1.2). Binary data are presented as numbers/total numbers and percent; continuous data are presented as medians (25th-75th percentile). For binary variables, their influence on glycocalyx shedding parameters has been examined by performing group comparisons employing Wilcoxon Rank Sum Tests. Adjustment for multiple testing was performed according to the Benjamini-Hochberg procedure. The relationship between continuous variables and glycocalyx shedding parameters was assessed by evaluating Pearson´s product-moment correlation coefficient. Stepwise changes over time in syndecan-1 and heparan sulfate were analyzed using a Friedman Rank Sum Test, followed by pairwise assessments employing a Wilcoxon Singed Rank Test with paired samples.

## Results

Thirty organs were included in the study. NMP was initiated for donor-related reasons in 15 cases, for logistical reasons in 13 cases, and for recipient-related reasons in 4 cases, with multiple indications applicable in several instances. No additional perfusion modalities (Hypothermic oxygenated perfusion or Normothermic regional perfusion) were used during procurement. In summary 88 timepoints were analyzed. Due to protocol violations regarding the sampling time points, one value at the end of NMP and one value on admission to ICU had to be excluded from analysis.

### Donor and Recipient Data

Donor data are presented in Table 1. Twenty-two grafts were from DBD donors and 8 from DCD donors. Nineteen donors were classified as extended criteria donors (ECD) [26]. Median cold ischemic time was 330 (262.26–438.25) minutes prior to perfusion, and NMP time was 1,058.5 (710.25–1,200) minutes. One graft was not transplanted due to extremely high transaminase levels. Two grafts were declined due to absent lactate clearance combined with an inability to maintain stable pH levels during perfusion. Two grafts were not used due absent lactate clearance and impaired parenchymal appearance. Two grafts were declined based solely on histopathological findings regarded as unsuitable for transplantation. One graft was not transplanted because of a malignancy diagnosed in the donor, and another transplantation was cancelled due to lymph node metastases of the allocated recipient. Characteristics of the 21 patients who were finally transplanted are presented in Table 2.

**TABLE 1:** Donor and Perfusion characteristics.

Age (years)	61 (5.8–70.3)
Sex	
Female	21 (70%)/30
Male	9 (30%)/30
BMI	26.5 (23–30)
Donor type	
DBD	22 (73.3%)/30
DCD	8 (26.7%)/30
Extended criteria donor	19 (63.3%)/30
DCD	8 (42.1%)/ 19
Donor ICU stay >7 days	3 (15.8%)/19
Donor age >65 years	9 (47.4%)/19
Elevated transaminases	4 (21.1%)/19
CS time (min)	330 (262.25–439.25)
NMP time (min)	1038.5 (710.25–1200)

**TABLE 2:** Recipient characteristics.

Age (years)	63.5 (48.75–67.25)
Sex (female/male)	6 (28.57%)/15 (71.43%)
Body weight (kg)	76.5 (62–83)
Body height (cm)	174.5 (166.5–178.5)
Meld score	15.5 (8.75–17.25)
Etiology of liver disease	
Alcohol	7 (33.3%)/21
Hepatocellular carcinoma	7 (33.3%)/21
Primary sclerotic cholangitis	3 (14.3%)/21
Polycystic liver disease	2 (9.5%)/21
Other	2 (9.5%)/21

Recipient´s median ICU and hospital length of stay were 3 (3–9) and 21 (17–37.25) days; 7 recipients required postoperative renal replacement therapy. Biliary complications, including anastomotic strictures, non-anastomotic strictures, bile leaks, episodes of cholangitis, and cholangiopathy, were observed in 7 recipients (33.3%), whereas 14 had none. Thirty-day and 90-day survival rates were 100% and 90.5%, respectively. Two early deaths occurred: One patient died 51 days after transplantation due to progressive graft dysfunction in acute rejection and 1 patient died on day 82 due to a cardiac event.

In total, 5 recipients died during the follow up period (median follow up: 23.7 months (19.7–27.8)).

### Glycocalyx Damage Parameters During NMP

#### Syndecan-1

**FIGURE 1:**
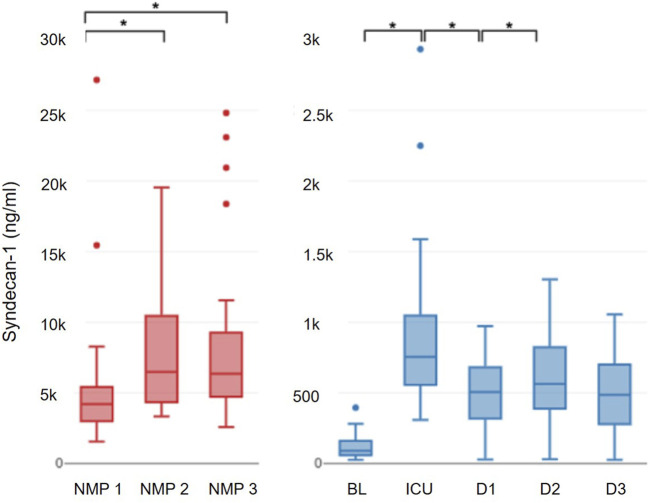
Progression of syndecan-1.

Syndecan-1 significantly increased during NMP, rising from 4,219.8 (2,999.3–5,414.8) ng/mL to 6,491.2 (4,338.9–10,468.2) ng/mL after 6 h (p = 0.001). There was no further increase in syndecan-1 until the end of NMP, with levels reaching 6,355.6 (4,720.2–9,260) ng/mL (p = 0.25; Figure 1).

In grafts from DCD donors, syndecan-1 levels were constantly higher throughout the entire NMP period compared to grafts from DBD donors (NMP 1: 5,337.96 ng/mL vs. 4,032.17 ng/mL, p = 0.031; NMP 2: 10,695.6 ng/mL vs. 5,801.39 ng/mL, p < 0.001; NMP 3: 9,727.55 ng/mL vs. 5,174.07 ng/mL, p = 0.025). Neither sex, laboratory parameters (AST, ALT, GGT, creatinine, leukocytes, CRP), nor pre-existing conditions (arterial hypertension, diabetes mellitus) of the donor correlated with the dynamics of syndecan-1 during NMP. No association between cold storage-time and syndecan-1 in perfusate could be shown. The classification as ECD appears to have no influence on syndecan-1 levels during NMP.

#### Heparan Sulfate

**FIGURE 2:**
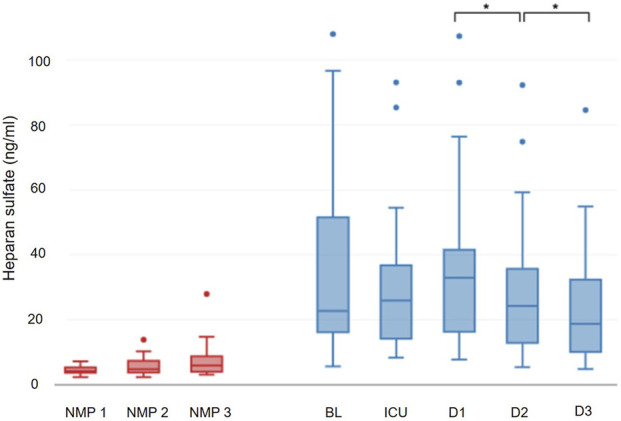
Progression of heparan sulfate.

Heparan sulfate levels were 4.2 (3.7–5.4) ng/mL after start of NMP and 4.8 (3.8–7.4) ng/mL after 6 h of NMP; at the end of NMP heparan sulfate concentrations were 5.95 (3.96–8.79) ng/mL. No significant dynamics of heparan sulfate were observed during NMP (p = 0.100) (Figure 2). No difference in heparan sulfate levels was detected between DBD and DCD donors during the first six hours of NMP. However, higher levels were measured in DCD grafts at the end of NMP (DBD: 5.23 (3.55–7.23) ng/mL vs. DCD: 8.99 (7.01–10.16) ng/mL; p = 0.047). No other recorded donor characteristics or laboratory parameter appeared to influence the level of heparan sulfate during NMP. Furthermore, no association was found between heparan sulfate levels in perfusate and the recipient´s postoperative course.

### Correlation Analysis of Syndecan-1 and Routine Perfusate Markers

Median perfusate glucose concentrations declined from 23.3 (17.65–28.5) mmol/L at the beginning of perfusion to 8.2 (5.07–11.85) mmol/L at its end. Perfusate sodium concentrations were 142 (134.75–143) mmol/L, 150 (142.75–156.5) mmol/L, and 150.5 (145.25–160) mmol/L at the beginning of perfusion, after 6 h, and at the end, respectively. No correlation between perfusate glucose or sodium and syndecan-1 measured at the same time points was observed (p = 0.138 for glucose; p = 0.95 for sodium).

Syndecan-1 showed a consistent association with established markers of hepatocellular injury. At the beginning of perfusion, syndecan-1 correlated with aspartate aminotransferase (AST) (r = 0.406, p = 0.04), with this relationship strengthening after 6 h (r = 0.51, p = 0.009) and persisting at the end of perfusion (r = 0.448, p = 0.026). Correlations with alanine aminotransferase (ALT) emerged at later time points, namely, after 6 h (r = 0.481, p = 0.014) and at the end of perfusion (r = 0.4, p = 0.049). A significant association with bilirubin was observed only at the end of perfusion (r = 0.488, p = 0.013). Syndecan-1 also correlated with lactate dehydrogenase throughout the perfusion course - at the start (r = 0.428, p = 0.03), after 6 h (r = 0.510, p = 0.009), and at the end (r = 0.468, p = 0.019). In contrast, neither pH nor lactate showed any correlation with syndecan-1.

### Outcome Associations of Glycocalyx Injury During NMP

**FIGURE 3:**
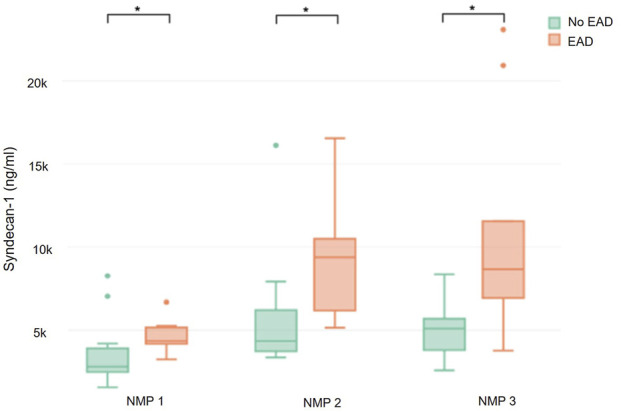
Syndecan-1 during NMP depending on the occurrence of EAD.

There was a significant correlation between syndecan-1 measured at the end of NMP and recipients AST levels on ICU admission (r = 0.55; p = 0.026) and the first three postoperative days (day 1: r = 0.58 p = 0.026; day 2: r = 0.48, p = 0.033; day 3: r = 0.51, p = 0.032). Furthermore, perfusate syndecan-1 values where significantly higher in grafts of recipients who developed EAD (n = 10) compared to those without EAD (n = 11) (4,331.5 (4,180.3–5,033.7) ng/mL vs. 2,800.7 (2,480.5–3,618.7) ng/mL; p = 0.024 and 9,379.7 (6,181–104,923) ng/mL vs. 4,338.9 (3,730–6,201.3) ng/mL; p = 0.013 and 8,663.6 (6,926.2–11,551.9) ng/mL vs. 5,096.5 (3,795–5,679.5) ng/mL; p = 0.013) (Figure 3). In contrast, syndecan-1 concentrations during NMP did not differ between recipients who subsequently developed acute kidney injury (AKI) grade II or III and those with no AKI or grade I.

**FIGURE 4:**
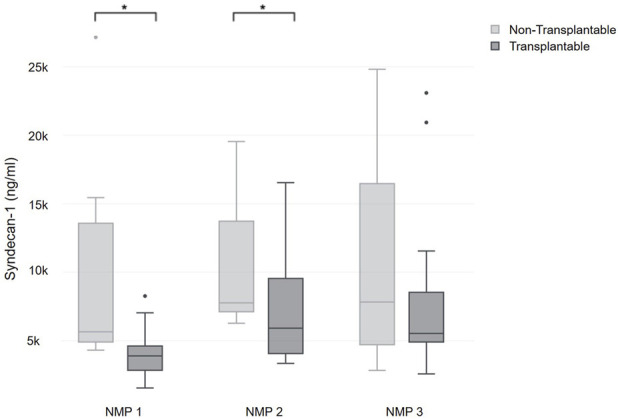
Comparison of syndecan-1 during NMP between organs classified as transplantable and non-transplantable.

Grafts that were classified as transplantable (n = 23) exhibited significantly lower syndecan-1 levels at the onset of perfusion (3,892.6 (2,883.7–4,561.2) ng/mL) and after six hours of perfusion (5,915.7 (4,082.2–9,379.7) ng/mL) when compared to grafts that were classified as unsuitable for transplantation due to their metabolic profile (5,653.7 (5,074–11,706.6) ng/mL; p = 0.001 and 7,764.8 (7,133.6–12,740.7) ng/mL; p = 0.037) (Figure 4) [24]. Of note, two grafts, which had been approved for transplantation based on metabolic performance, were not transplanted due to the presence of malignancies in the donor or recipient.

### Glycocalyx Damage Parameters in the Recipient

Syndecan-1 levels in recipients blood significantly increased from baseline (90.69 (58–161) ng/mL) to ICU admission (755.87 (556.3–1,046.8); p < 0.001), followed by a notable decline on the first (563.3 (389–822) ng/mL; p = 0.008) and second (506.3 (319–680) ng/mL; p = 0.002) postoperative day. Between the second and the third postoperative day, syndecan-1 levels remained constant (485.8 (278.75–699.3) ng/mL; p = 0.366) (Figure 1). From postoperative day 1 onwards, syndecan-1 concentrations differed significantly between recipients who developed no or grade I AKI compared to those who progressed to AKI grade II or III (404.8 (227.7–529.4) ng/mL vs. 790.9 (562–1,108.9) ng/mL; p = 0.01); this divergence persisted through postoperative day 3 (322 (85.4–408.7) ng/mL vs. 691.7 (586.6–832.5) ng/mL; p = 0.003).

In contrast, recipients plasma levels of heparan sulfate did not increase during transplantation (25.9 (14.6–36.7) ng/mL; p = 0.508) or on postoperative day 1 (32.9 (17.6–41.3) ng/mL; p = 0.926). However, from postoperative day 1 to postoperative day 2 (24.3 (13.2–35.1) ng/mL; p = 0.006) and postoperative day 3 (18.7 (10.1–30.8) ng/mL; p = 0.008), heparan sulfate levels experienced a decrease, reaching values below the preoperative baseline (p = 0.006) (Figure 2).

## Discussion

The main finding of this prospective observational study was that the degree of glycocalyx alteration during NMP is predictive for EAD after orthotopic liver transplantation. Furthermore, grafts that were ultimately classified as non-transplantable exhibited significantly higher syndecan-1 levels at the start of perfusion in comparison to those that were released for transplantation. Syndecan-1 and heparan sulfate both increased during NMP, indicating ongoing glycocalyx damage. Not surprising, the glycocalyx of DCD organs appeared to be more severely damaged compared to DBD grafts, exhibiting significantly higher syndecan-1 and heparan sulfate levels compared to DBD grafts. Elevated syndecan-1 levels decreased postoperatively; heparan sulfate levels were comparable to the patients’ preoperative baseline and decreased even further during the first two postoperative days.

Despite the fact that NMP is already in widespread clinical use, a significant number of pathophysiological processes during NMP have not yet been investigated. The present study provides preliminary evidence of progressive damage to the endothelial glycocalyx, as indicated by increased glycocalyx components in perfusate.

Two hypotheses may be introduced to explain these findings: First, the introduction of NMP results in the establishment of a non-physiological laminar flow; whereas pulsatile flow seems to protect glycocalyx integrity, laminar flow seems to be damaging [27, 28].

Second, the composition of the perfusate may play a role in maintaining glycocalyx stability [29, 30]. It was shown that fresh frozen plasma and various plasma components positively influence glycocalyx integrity by promoting endothelial stabilization [31, 32]. In addition, animal studies indicate a positive effect of hydroxyethyl starch (HES 130/0.4) a synthetic colloid [33, 34]. Whether this effect extends to gelatin, the colloid used in the perfusion fluid of our study, remains uncertain.

A further major component of the perfusate are red blood cells. In a study on hemorrhaged rats investigating the effects of resuscitation with blood products on the microcirculation, unwashed red blood cells have been shown to restore the glycocalyx [35]. However, this effect was no longer observed when the red blood cells were washed and all residual plasma components removed. The authors concluded that the effects of unwashed erythrocytes on the glycocalyx are primarily attributable to residual plasma components, rather than red blood cells themselves [35].

The specific biochemical environment within the perfusate may also affect glycocalyx stability, particularly regarding to glucose, sodium, and oxygen levels: Marked hyperglycemia, hypernatremia, and hyperoxia were observed during *ex situ* perfusion, each representing a distinctly unphysiological condition. Experimental and clinical studies have demonstrated that excessive glucose exposure, elevated sodium concentrations, and supraphysiological oxygen tensions can induce structural disruption, shedding, or functional impairment of the endothelial glycocalyx [36–38]. It is therefore plausible that the constellation of these factors during perfusion may have amplified the extent of glycocalyx damage detected in the present cohort.

Further studies are needed to investigate the effects of different perfusion fluid components on the integrity or restoration of the endothelial glycocalyx.

The observed correlations between syndecan-1 and established markers of hepatocellular injury suggest that glycocalyx disruption represents a distinct yet integrative component of graft injury during NMP. Syndecan-1 release reflects damage to the endothelial barrier, a microstructural interface that differs fundamentally from hepatocellular injury captured by AST, ALT, bilirubin, or LDH [39]. The consistent association between syndecan-1 and these conventional markers indicates that endothelial glycocalyx loss accompanies and may contribute to broader cellular injury processes within the graft. By identifying this vulnerable structural layer as a quantifiable element of perfusion-related damage, the present findings highlight a potentially actionable target for future interventions. Strategies aimed at preserving glycocalyx integrity during NMP could therefore serve as a complementary approach to reducing overall graft injury.

A central finding of this study was that the levels of syndecan-1 at the end of NMP correlated with the development of EAD. This finding is in line with previous studies in which levels of syndecan-1 measured in graft efflux during the flushing phase after SCS were associated with the occurrence of EAD [19]. Our study demonstrates that during the course of NMP, a noticeable difference in glycocalyx damage can be observed between grafts that develop EAD and those that do not. However, it must be acknowledged that the sample size is limited, and the discriminatory ability may be overstated. While these associations are noteworthy, the study was not designed to evaluate long-term graft function or to propose glycocalyx markers as decision-making tools for graft acceptance. Rather, the primary intention was to characterize a vulnerable endothelial structure that remains poorly explored in the context of NMP and to assess whether its injury profile is associated with early postoperative trajectories. The observed correlations therefore underscore that glycocalyx disruption during NMP may reflect a biologically meaningful process, supporting further mechanistic research and the development of perfusion strategies aimed at protecting this important microvascular interface.

In the present cohort, syndecan-1 concentrations did not differ during NMP between recipients who later developed AKI grade II–III and those with no or only grade I AKI; however, from postoperative day 1 to day 3, markedly higher syndecan-1 levels were observed in those who progressed to AKI grade II–III. This postoperative pattern is consistent with observations previously reported by Schiefer et al [20]. The temporal separation emerging only after transplantation suggests that postoperative systemic factors, including bleeding, hemodynamic instability, and inflammatory activation, are linked to both glycocalyx degradation and renal dysfunction, indicating a shared pathophysiological background rather than a direct causal relationship [40, 41]. These observations reinforce the notion that postoperative endothelial vulnerability represents a relevant pathophysiological mechanism and may constitute a target for perioperative strategies aimed at mitigating AKI following liver transplantation [42].

The observation that grafts ultimately not used for transplantation exhibited elevated syndecan-1 levels in the perfusate at the commencement of NMP could suggest that this value may serve as a predictor of the future metabolic performance of the graft at an early stage of perfusion. The provision of earlier information on the condition of the organ could prove advantageous in two respects. Firstly, it could facilitate the resolution of logistical challenges. Secondly, it could initiate any therapies related to the graft during NMP, a subject that is currently the focus of intensive research, at an earlier stage [43].

It is noteworthy that the course of heparan sulfate levels markedly diverged from that of syndecan-1: During NMP heparan sulfate levels showed no dynamics. Following admission to ICU heparan sulfate levels were comparable to the recipients’ baseline and even decreased over the next 3 days, reaching levels that were even lower than baseline. As syndecan-1 carries heparan sulfate as side chains, it could be assumed that cleavage of syndecan-1 would simultaneously lead to cleavage of heparan sulfate. Interestingly, in our data, heparan sulfate levels only increased at the end of NMP but not at any other time point. Similar discrepancies have been reported in other studies where both glycocalyx markers were measured [44, 45]. Passov et al. extensively investigated the dynamics of heparan sulfate levels in the context of liver transplantation [46]. They observed lower heparan sulfate levels in the efflux of the graft, compared to levels in the portal vein; Passov proposed that heparan sulfate may be reabsorbed into the graft. It is currently unclear whether this finding represents a glycocalyx restoration mechanism. However, cell culture experiments support this hypothesis [47]. Nevertheless, postoperative heparan sulfate values below baseline may also indicate enhanced liver function after transplantation compared to the recipient’s preoperative condition. In fact, Schiefer et al. demonstrated that glycocalyx components in patients scheduled for liver transplantation were markedly elevated compared to a healthy control population [20].

The results of this study point towards new possibilities for maintaining physiological functioning of the graft during NMP. In recent years several substances with possible glycocalyx restoring characteristics have been identified [48]. NMP offers the advantage of safely adding these substances to the perfusate. Data regarding these new possibilities, however, are still lacking.

Glycocalyx shedding has already been described in liver grafts after SCS. Schiefer et al. demonstrated significantly increased levels of syndecan-1 in the perfusion fluid of SCS grafts right before transplantation [26]. However, our study differs from this previous study, in that SCS was followed by NMP. It is evident that a direct comparison between the results of this work an those of previously published studies is not feasible. However, it is pertinent to acknowledge that despite similar baseline values, syndecan-1 levels at ICU admission were significantly lower in our study compared to a previous study of SCS graft recipients [19]. This finding may be attributed to the fact that in NMP grafts the very “first” reperfusion occurs with start of NMP; in SCS grafts however, reperfusion entirely happens in the recipient. It could be hypothesized that reperfusion occurring in the machine is more controlled compared to the intraoperative setting where bleeding and hemodynamic perturbations may pose a further challenge.

Our study has several limitations. First, the number of grafts analyzed was small, with 30 grafts included in the study. The small number of grafts and the heterogeneity of the cohort may introduce substantial bias and limit the generalizability of the findings; therefore, this limitation must be explicitly acknowledged, and the results should be interpreted with caution. This study was conceived with the objective of investigating the phenomenon of glycocalyx destruction during normothermic machine perfusion. However, the extent of glycocalyx destruction during machine perfusion appears to have a greater influence on the postoperative outcome of recipients than was previously assumed. Even though the study was not designed to establish a correlation between glycocalyx damage and the outcomes of the recipients, and that the exploratory data analysis was primarily intended to generate further hypotheses, the results were so interesting, that they have been included in the present publication. A further limitation is the detection of the endothelial glycocalyx itself. Direct imaging of the glycocalyx would be advantageous; however, this is a challenging procedure due to its vulnerable nature and was not feasible in the present study; the measurement of shedded glycocalyx components is a commonly employed approach and accepted as a surrogate marker instead. A further constraint arises from the limited size of the transplanted patient cohort, which renders long-term outcome measures, such as overall survival, difficult to interpret and highly susceptible to the influence of single individual outcomes, however, analyses from the same center have demonstrated that overall mortality is generally within the expected range [21]. In future studies, the intervals utilized for the analysis of parameters during NMP may benefit from more precise alignment. This would facilitate the creation of a more precise representation of the course and would ensure that already falling values are not misinterpreted as peaks.

In summary, our data show that the endothelial glycocalyx is damaged during NMP liver transplantation. Shedding of glycocalyx proteins appears to be particularly pronounced in grafts derived from DCD donors. Furthermore, in the patient cohort under investigation, higher levels of glycocalyx shedding parameters during NMP significantly correlated with the development of EAD. This correlation was detected as early as 6 h after start of NMP which could potentially impact future donor recipient decisions. However, given the limited sample size and the heterogeneity of the studied cohort, the findings must be interpreted with caution. Further studies with larger and more homogeneous populations are required to validate these observations and to determine their clinical relevance.

## Data Availability

The raw data supporting the conclusions of this article will be made available by the authors, without undue reservation.
