# Correction: Effect of normothermic machine perfusion on glycocalyx shedding during liver transplantation - a prospective pilot study

**DOI:** 10.3389/ti.2026.16694

**Published:** 2026-05-06

**Authors:** Simon Mathis, Gabriel Putzer, Lukas Gasteiger, Nikolai Staier, Lisa Schlosser, Pia Tscholl, Robert Breitkopf, Benno Cardini, Alexander Kofler, Rupert Oberhuber, Thomas Resch, Stefan Schneeberger, Judith Martini

**Affiliations:** 1 Department of Anaesthesiology and Critical Care Medicine, Medical University of Innsbruck, Innsbruck, Austria; 2 Department of Visceral, Transplant and Thoracic Surgery, Medical University of Innsbruck, Innsbruck, Austria; 3 Data Lab Hell GmbH, Zirl, Austria

**Keywords:** glycocalyx, heparan sulfate, liver transplantation, normothermic machine perfusion, syndecan-1

There was a mistake in Figures 1–3 as published. The figures were placed in the wrong order in the text. The corrected Figures 1–3 appears below.

**FIGURE 1:**
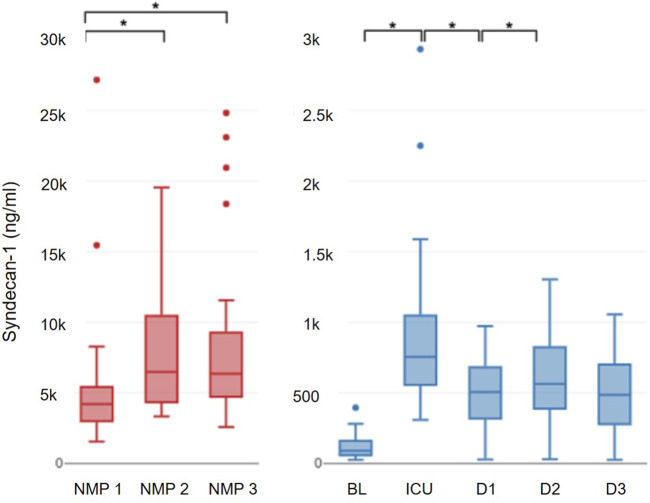
Progression of syndecan-1.

**FIGURE 2:**
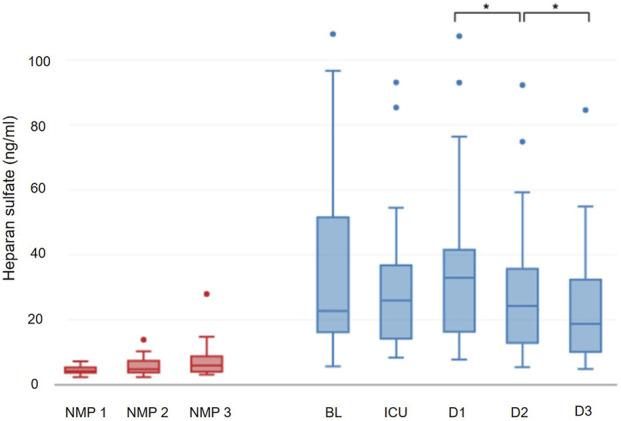
Progression of heparan sulfate.

**FIGURE 3:**
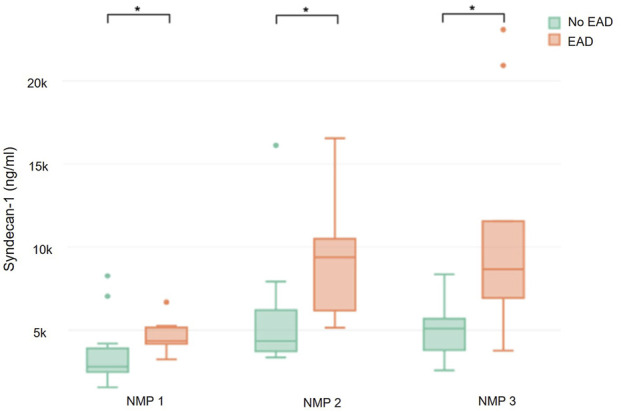
Syndecan-1 during NMP depending on the occurrence of EAD.

There was a mistake in the caption of Figure 2 as published. The corrected caption of Figure 2 appears below.

There was a mistake in the caption of Figure 3 as published. The corrected caption of Figure 3 appears below.

There was a mistake in the caption of Figure 4 as published. The corrected caption of Figure 4 appears below.

**FIGURE 4:**
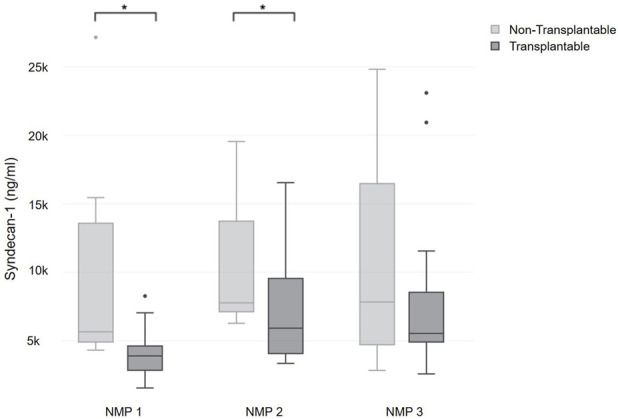
Comparison of syndecan-1 during NMP between organs classified as transplantable and non-transplantable.

A correction has been made to the section **Results**, Sub Section “*Glycocalyx Damage Parameters during NMP”*, Subsub Section “*Heparan Sulfate*”, Paragraph “1”. The Sentence: “No significant dynamics of heparan sulfate were observed during NMP (p = 0.100) **(** Figure 4)” has been changed to: “No significant dynamics of heparan sulfate were observed during NMP (p = 0.100) (Figure 2).”

A correction has been made to the section Section “**Results**”, Sub Section “*Outcome Associations of Glycocalyx Injury During NMP*”, Paragraph “1”. The Sentence: “Furthermore, perfusate syndecan-1 values where significantly higher in grafts of recipients who developed EAD (n = 10) compared to those without EAD (n = 11) (4,331.5 (4,180.3–5,033.7) ng/mL vs. 2,800.7 (2,480.5–3,618.7) ng/mL; p = 0.024 and 9,379.7 (6,181–104,923) ng/mL vs. 4,338.9 (3,730–6,201.3) ng/mL; p = 0.013 and 8,663.6 (6,926.2–11,551.9) ng/mL vs. 5,096.5 (3,795–5,679.5) ng/mL; p = 0.013) (Figure 2).” should be changed to: “Furthermore, perfusate syndecan-1 values where significantly higher in grafts of recipients who developed EAD (n = 10) compared to those without EAD (n = 11) (4,331.5 (4,180.3–5,033.7) ng/mL vs. 2,800.7 (2,480.5–3,618.7) ng/mL; p = 0.024 and 9,379.7 (6,181–104,923) ng/mL vs. 4,338.9 (3,730–6,201.3) ng/mL; p = 0.013 and 8,663.6 (6,926.2–11,551.9) ng/mL vs. 5,096.5 (3,795–5,679.5) ng/mL; p = 0.013) (Figure 3).”

A correction has been made to the section Section “**Results**”, Sub Section “*Outcome Associations of Glycocalyx Injury During NMP*”, Paragraph “2”. The Sentence: “Grafts that were classified as transplantable (n = 23) exhibited significantly lower syndecan-1 levels at the onset of perfusion (3,892.6 (2,883.7–4,561.2) ng/mL) and after six hours of perfusion (5,915.7 (4,082.2–9,379.7) ng/mL) when compared to grafts that were classified as unsuitable for transplantation due to their metabolic profile (5,653.7 (5,074–11,706.6) ng/mL; p = 0.001 and 7,764.8 (7,133.6–12,740.7) ng/mL; p = 0.037) (Figure 3).” should be changed to “Grafts that were classified as transplantable (n = 23) exhibited significantly lower syndecan-1 levels at the onset of perfusion (3,892.6 (2,883.7–4,561.2) ng/mL) and after six hours of perfusion (5,915.7 (4,082.2–9,379.7) ng/mL) when compared to grafts that were classified as unsuitable for transplantation due to their metabolic profile (5,653.7 (5,074–11,706.6) ng/mL; p = 0.001 and 7,764.8 (7,133.6–12,740.7) ng/mL; p = 0.037) (Figure 4).”

A correction has been made to the section Section “**Results**”, Sub Section “*Glycocalyx Damage Parameters in the Recipient*”, Paragraph “2”. The Sentence: “However, from postoperative day 1 to postoperative day 2 (24.3 (13.2–35.1) ng/mL; p = 0.006) and postoperative day 3 (18.7 (10.1–30.8) ng/mL; p = 0.008), heparan sulfate levels experienced a decrease, reaching values below the preoperative baseline (p = 0.006) (Figure 4).” should be changed to “However, from postoperative day 1 to postoperative day 2 (24.3 (13.2–35.1) ng/mL; p = 0.006) and postoperative day 3 (18.7 (10.1–30.8) ng/mL; p = 0.008), heparan sulfate levels experienced a decrease, reaching values below the preoperative baseline (p = 0.006) (Figure 2).”

The original article has been updated.

## Generative AI statement

Any alternative text (alt text) provided alongside figures in this article has been generated by Frontiers with the support of artificial intelligence and reasonable efforts have been made to ensure accuracy, including review by the authors wherever possible. If you identify any issues, please contact us.

